# Coronary perforation during insertion of a long stent in a severely calcified lesion

**DOI:** 10.1002/ccr3.4217

**Published:** 2021-07-06

**Authors:** Yoshinobu Murasato, Kyohei Meno, Takahiro Mori, Katsuhiko Takenaka

**Affiliations:** ^1^ Department of Cardiology and Clinical Research Center National Hospital Organization Kyushu Medical Center Fukuoka Japan

**Keywords:** complication, percutaneous coronary intervention, stents

## Abstract

We reported a case that the insertion of a 48‐mm‐long stent in a calcified coronary lesion after rotational atherectomy led to stent stacking and S‐shaped flection, resulting in longitudinal coronary perforation without stent inflation. Its flexibility and length pose a possible risk of deformation inside the vessel during stent insertion.

## Graphical abstract

Insertion of a 48‐mm‐long stent after rotational atherectomy led to stent stacking and S‐shaped flection, resulting in coronary perforation without stent inflation.

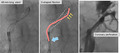

## 1 INTRODUCTION

The use of a long stent to treat diffuse complex lesions decreases the number of stents needed and thus their overlapping. However, its flexibility and length pose a possible risk of deformation inside the coronary artery leading to coronary perforation during stent insertion.

## 2 CASE REPORT

**FIGURE 1:**
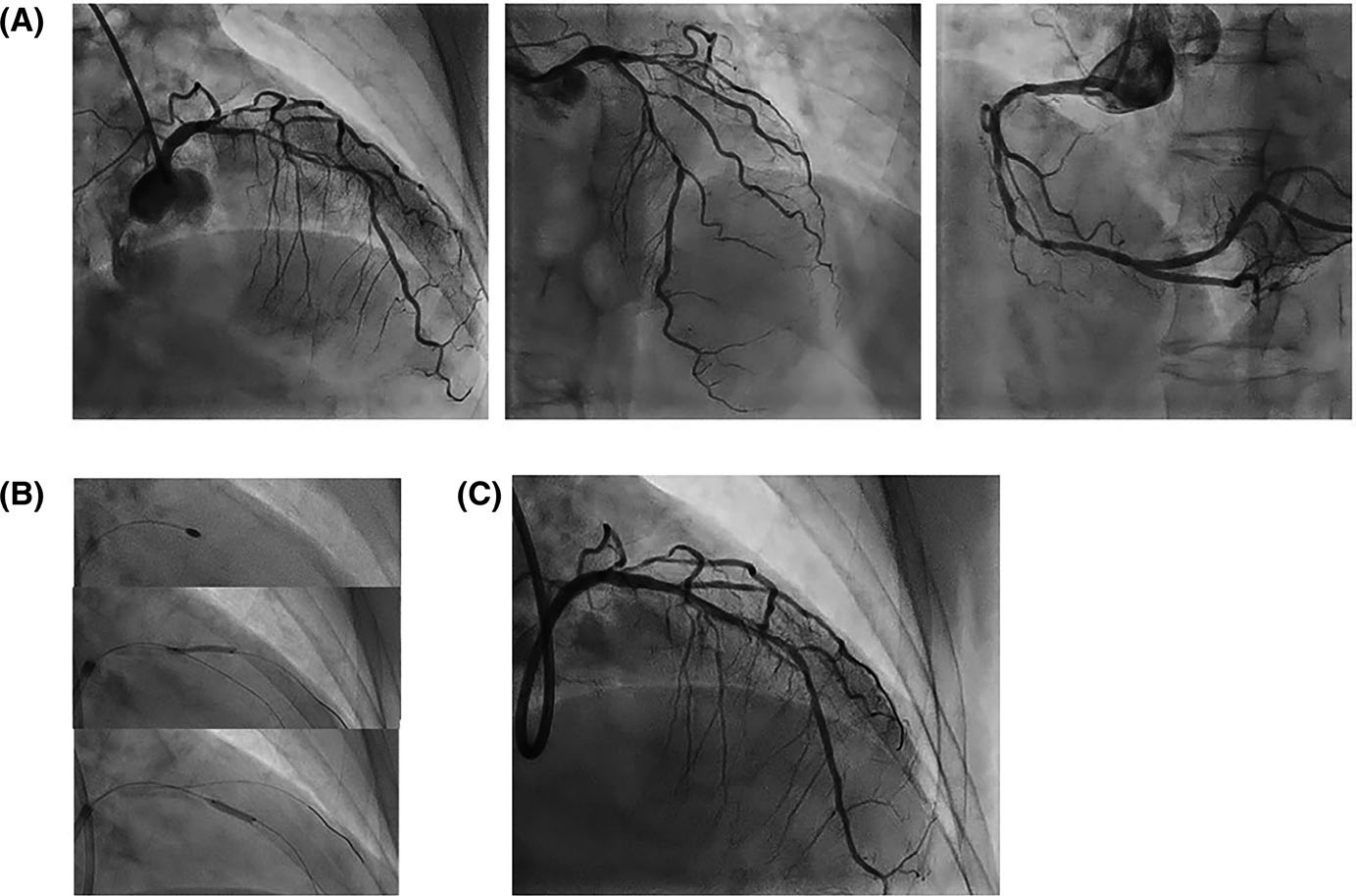
A, Baseline coronary angiography (CAG): (left and middle) left coronary artery; (right) right coronary artery. B, Coronary intervention: (top) rotational atherectomy in left anterior descending artery (LAD); (middle) 2.0‐mm‐diameter balloon dilation in diagonal branch; (lower) 2.5‐mm‐diameter cutting balloon dilation in LAD. C. CAG before stent insertion

The patient was a 74‐year‐old woman with unstable angina and comorbidities of hypertension, diabetes mellitus, and dyslipidemia. Coronary angiography (CAG) showed a tight diffuse calcified lesion in the left anterior descending artery (LAD), a 1‐1‐1 bifurcation lesion between the diagonal branch (DB), and a moderate diffuse lesion in the right coronary artery (Figure 1A, Videos S1 and S2).

**FIGURE 2:**
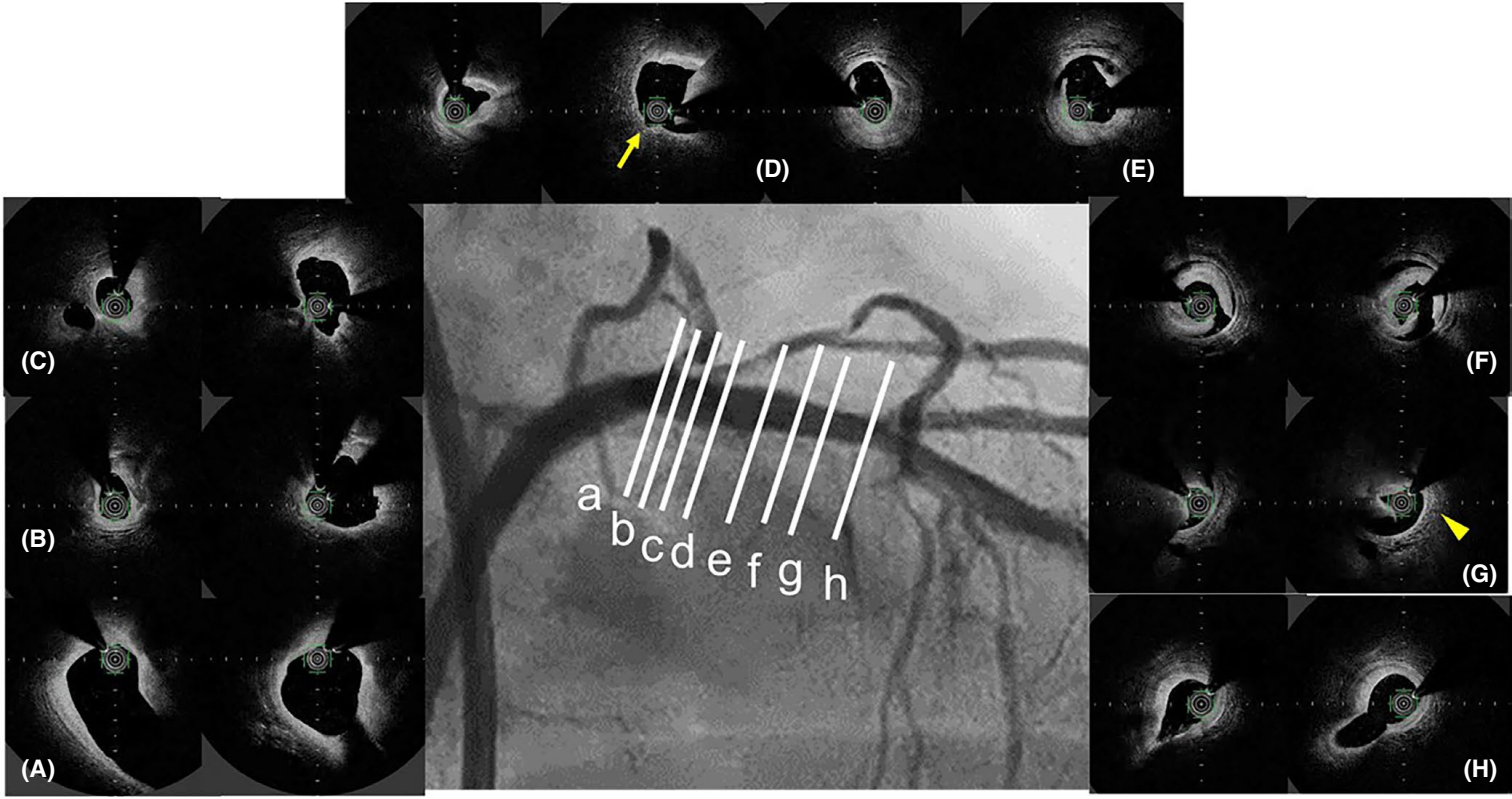
Optical frequency domain imaging (OFDI) images immediately after first rotational atherectomy with a 1.5‐mm burr (left) and just before stent insertion (right). Each panel (A‐H) corresponds to the line in the center coronary angiogram. Dilation with a cutting balloon following rotational atherectomy led to adequate lumen expansion (B‐E) and intimal injury by the cutting balloon blade (D, arrow). However, eccentric calcification remained in the distal site with the OFDI catheter deviated to the healthy site (G, triangle)

**FIGURE 3:**
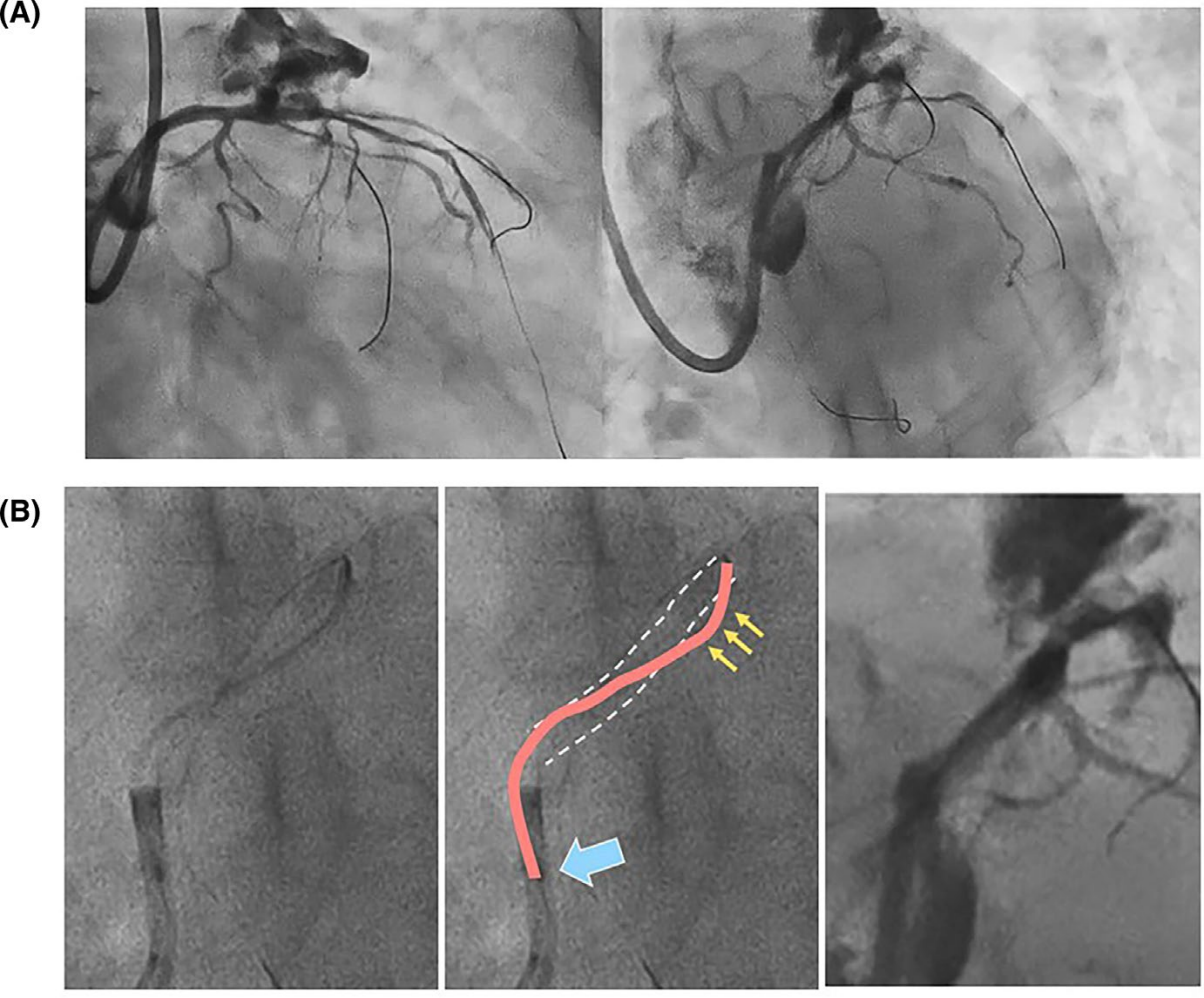
Coronary perforation during stent insertion. A (right) Right anterior oblique cranial view, (left) left anterior oblique caudal view. B (left) Magnified view of the S‐shaped flection of the 48‐mm‐long stent, (middle) scheme of the coronary artery (dotted line) and S‐shaped stent (orange line). Proximal one‐third of the stent remained in the guiding catheter (arrow) and the distal flection curve of the stent was outside of the vessel (arrows); (right) coronary perforation occurred at the distal flection site

We performed coronary intervention for lesions in the LAD and DB using a 7‐Fr system via the radial artery. Severe calcified lesion was ablated using the Rotablator™ Rotational Atherectomy System (Boston Scientific, Natick, MA, USA) with 1.5‐ and 1.75‐mm burrs at 180,000 rpm, guided by optical frequency domain imaging (OFDI; FastView™ catheter, Terumo Corp., Tokyo, Japan). Then, the LAD and DB were dilated using a 2.5/10‐mm cutting balloon (WOLVERINE™, Boston Scientific) at 12 atm and a 2.0/15‐mm semi‐compliant balloon, respectively (Figure 1B, C). OFDI demonstrated that ablation and lumen expansion were sufficient to deploy the stent (Figure 2, Video S3). To avoid compromising the DB, the jailed balloon technique was performed using a 2.5/48‐mm Xience Xpedition™ stent (Abbott) for the LAD and a 2.0/15‐mm balloon for the DB. However, the LAD stent encountered resistance and could not be advanced. After retrieving the DB balloon, we attempted to reinsert the LAD stent using both right anterior oblique (RAO) and left anterior oblique (LAO) views. Although the stent seemed to advance straight to the distal part of the RAO cranial view (Video S4), the S‐shaped flection of the stent was apparent in the LAO caudal view (Video S5). Systolic blood pressure suddenly dropped to less than 80‐mmHg despite the pre‐inflation of the stent, and the CAG showed a large coronary perforation (Figure 3, Videos S6 and S7).

**FIGURE 4:**
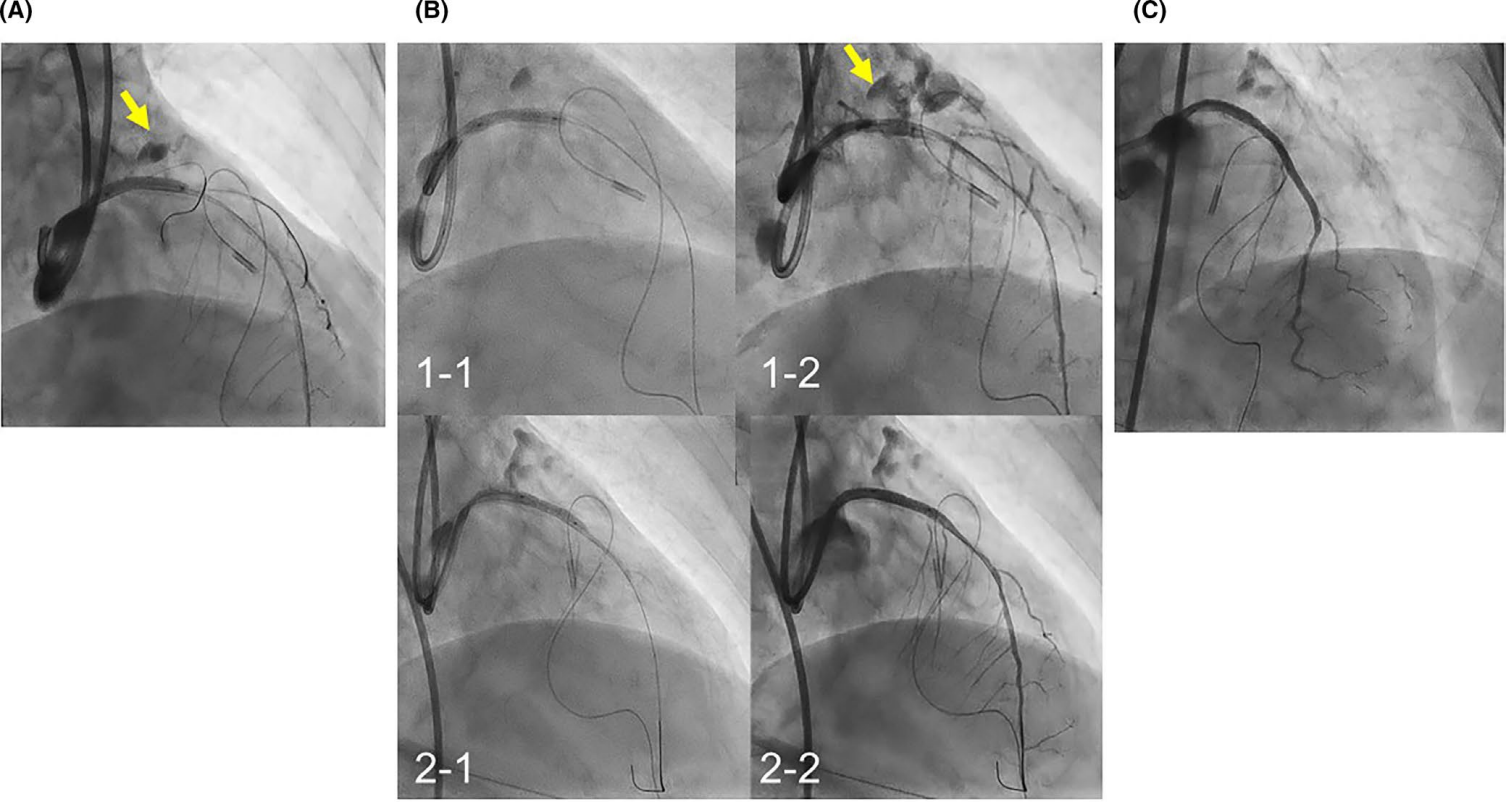
A, Dilation with a 3.0‐mm‐diameter perfusion balloon failed to obtain hemostasis (arrow). B, Implantation of a 3.5/16‐mm covered stent (1‐1) also failed to obtain hemostasis (1‐2, arrow). Subsequent implantation of a 2.8/16‐mm covered stent (2‐1) accomplished hemostasis (2‐2). C, Final coronary angiography showed loss of circumflex artery and first diagonal branch

The stent was immediately retrieved, and a 2.5‐mm perfusion balloon was inflated at the perforation site; large extravasation remained. Another 7‐Fr guiding system was inserted via the right femoral artery, and a 3.0‐mm perfusion balloon was inflated after retrieving the 2.5‐mm balloon. Since neither a 2.5‐mm nor a 3.0‐mm perfusion balloon could complete hemostasis (Figure 4A), cardiocentesis was performed. Serious hemodynamic collapse required subsequent insertion of an extracorporeal membrane oxygenation system. Hemostasis was completed after consecutive implantation of 3.5/16‐mm and 2.8/16‐mm covered stents (Graftmaster, Abbott) (Figure 4B). An additional stent (Synergy 2.5/28 mm, Boston Scientific) was deployed at the distal pretreated site (Video S8).

The patient survived despite acute myocardial infarction due to the sacrifice of the hypoplastic left circumflex artery and DB, and all mechanical support systems were extracted within a week. However, heart failure and multiple organ failure progressed gradually, and the patient died 1 month later.

## 3 DISCUSSION

In this case, a large longitudinal coronary perforation occurred during the insertion of a 48‐mm‐long stent in a calcified lesion without any stent inflation. The following mechanisms were possibly considered: (a) Coronary perforation caused by the tip of the stent with an excessive forward power, (b) Delayed coronary perforation after rotational atherectomy followed by cutting balloon inflation, and (c) Longitudinal tear of coronary vessel due to a long stent stacking and S‐shaped flection. The first mechanism was unlikely since there was no protrusion of the stent or guidewire to the outer side of the vessel. The second mechanism was also unlikely because this type of perforation would have presented oozing. However, a large vessel tear with significant extravasation occurred during the stent insertion. As for the third mechanism, the fluoroscopy clearly demonstrated that the insertion of a 48‐mm‐long stent in a calcified lesion led to stent stacking and S‐shaped flection (Figure 3 and Video S5) resulting in a large longitudinal coronary perforation without stent inflation. OFDI performed before coronary perforation showed adequate calcium ablation and sufficient lumen enlargement in the proximal site (Figure 2A−D) and medial dissection in the distal site (E‐G). The healthy intima was injured with the cutting balloon at the perforation site (D, arrow). The stent tip might be stacked in the remaining eccentric calcification (G, triangle), and the dissected space (E‐G) assisted making a wide S‐shaped stent flection over the diameter of the native coronary vessel caused longitudinal coronary tear proximally from the stent stacking site on the contralateral site of eccentric calcification. In addition, one‐third of the stent remained in the guiding catheter, which possibly induced strong push ability to contribute on making S‐shaped flection in the coronary artery.

When we insert a long stent in the eccentric calcified lesion accompanied with dissection after pretreatment, special attention should be paid. Longer stent has more risk of trapping the proximal site of stent in the guiding catheter, which may enhance more push ability to make the stent flection inside a coronary vessel. In order to avoid this complication, consider performing one of the following before insertion: (a) biplane fluoroscopy for detailed observation of stent stacking due to failure to detect stent flection in the tangential view (Video S4 vs S5), (b) buddy wire technique to change the guidewire bias, or (c) insertion of a guide extension catheter to enhance the backup force and prevent stent flection inside the coronary artery.

In previous studies, the incidence of coronary perforation was 0.37%‐0.42% of all coronary interventions,^1^, ^2^, ^3^ with 65%‐74% of the cases occurring after stent and/or balloon inflation,^1^, ^2^, ^3^ 0.8%‐2.7% after cutting balloon inflation,^1^, ^2^ 1.7% after rotational atherectomy,^2^ and the remainder after guidewire perforation.^1^, ^2^ Although the use of a cutting balloon and rotational atherectomy is considered possible risk factors for coronary perforation, multivariate analysis has not identified them as independent risk factors.^1^, ^2^, ^3^ To the best of our knowledge, this is the first report of coronary perforation occurring during stent insertion, where the S‐shaped flection of the stent inside the coronary artery led to a longitudinal tear of the vessel.

## 4 CONCLUSION

The insertion of a 48‐mm‐long stent in a calcified lesion after rotational atherectomy led to stent stacking and S‐shaped flection, resulting in a large longitudinal coronary perforation without stent inflation. Biplane fluoroscopy was useful to identify the stent deformation. Buddy wire technique or insertion of a guide extension catheter should be considered in case of the resistance during the insertion of a very long stent.

## CONFLICT OF INTEREST

The authors have no conflicts of interest to declare.

## AUTHORS CONTRIBUTIONS

YM is a main operator who performed the coronary intervention, and a primary author of manuscript development. KM, TM, and KT supported the coronary intervention, collected background data, and provided significant authorship in the development of manuscript.

## ETHICAL APPROVAL AND CONSENT TO PARTICIPATE

Patient signed the informed consent document and accepted the publication of clinical data for research and scientific purposes. This treatment strategy has been approved by the institutional ethics review board.

## Supporting information

Video S1

Video S2

Video S3

Video S4

Video S5

Video S6

Video S7

Video S8

Supplementary Material

## Data Availability

All data generated or analyzed during this study are included in this published article.
