# Supplementary material for: Coronary perforation during insertion of a long stent in a severely calcified lesion
Source: Clin Case Rep. 2021 Jul 6;9(7):e04217. doi: 10.1002/ccr3.4217 (PMC8259801; doi:10.1002/ccr3.4217)
Supplement: Supplementary file 9 — Supplementary Material [file CCR3-9-e04217-s005.docx]

**Supplement**

**Supplement Movie 1:** Baseline left coronary angiography (CAG), right anterior oblique (RAO) cranial view.

**Supplement Movie 2:** Baseline left CAG, left anterior oblique (LAO) cranial view.

**Supplement Movie 3:** Optical frequency-domain imaging immediately after rotational atherectomy with a 1.5mm burr (left panel) and just before stent insertion (right panel).

**Supplement Movie 4:** Fluoroscopy in RAO cranial view during insertion of a 48mm-length stent.

**Supplement Movie 5:** Fluoroscopy in LAO caudal view during insertion of a 48mm-length stent.

**Supplement Movie 6:** Coronary perforation in RAO cranial view. DB: diagonal branch

**Supplement Movie 7:** Coronary perforation in LAO caudal view. DB: diagonal branch

**Supplement Movie 8:** Final coronary angiography.
